# High Inequalities Associated With Socioeconomic Deprivation in Cardiovascular Disease Burden and Antihypertensive Medication in Hungary

**DOI:** 10.3389/fphar.2018.00839

**Published:** 2018-08-03

**Authors:** Klára Boruzs, Attila Juhász, Csilla Nagy, Zoltán Szabó, Mihajlo Jakovljevic, Klára Bíró, Róza Ádány

**Affiliations:** ^1^Department of Health Systems Management and Quality Management in Health Care, Faculty of Public Health, University of Debrecen, Debrecen, Hungary; ^2^Department of Public Health, Government Office of Capital City Budapest, Budapest, Hungary; ^3^Department of Emergency Medicine, Medical Center, University of Debrecen, Debrecen, Hungary; ^4^Department of Global Health, Economics & Policy, Faculty of Medical Sciences, University of Kragujevac, Kragujevac, Serbia; ^5^MTA-DE Public Health Research Group of the Hungarian Academy of Sciences, Department of Preventive Medicine, Faculty of Public Health, University of Debrecen, Debrecen, Hungary

**Keywords:** antihypertensive drugs, cardiovascular mortality, deprivation, primary care, primary non-compliance, prescription, redemption

## Abstract

The wide life expectancy gap between the old and new member states of the European Union is most strongly related to the high rate of premature mortality caused by cardiovascular diseases (CVDs). To learn more about the background of this gap, the relationship of socioeconomic status (SES) with CVD mortality, morbidity and the utilization of antihypertensive drugs was studied in Hungary, a Central-Eastern European country with an extremely high relative risk of premature CVD mortality. Risk analysis capabilities were used to estimate the relationships between SES, which was characterized by tertiles of a multidimensional composite indicator (the deprivation index) and CVD burden (mortality and morbidity) as well as the antihypertensive medications at the district level in Hungary. The excess risks caused by premature mortality from CVDs showed a strong correlation with deprivation using the Rapid Inquiry Facility. The distribution of prevalence values related to these diseases was found to be similar, but in the areas of highest deprivation, where the prevalence of chronic ischaemic heart diseases and cerebrovascular diseases was found to be higher than the national average by 30 and 20%, the prevalence of hypertension exceeded the national average by only 4%. A linear association between the relative frequency of prescriptions/redemptions and deprivation for most antihypertensive drugs, except angiotensinogen receptor blockers, was shown. More intense screening for hypertension is proposed to improve the control of CVDs in countries affected by high disease burden.

## Introduction

The Marmot's report (European Commission, 2013) and the latest joint publication of the OECD and the European Commission (OECD and EU, 2016) on health and access to health services demonstrate that significant health inequalities exist between and within EU member states. The health status of the populations of the member states that joined the European Union in 2004 and after (EU13 countries) is less favorable than that of the countries that became members before 2004 (EU15 countries). Regarding particular causes of death, the largest contributor to mortality differences is cardiovascular diseases. Although the mortality caused by cardiovascular diseases (CVDs) is continuously decreasing, the relative risk of premature death, i.e., the ratio between the early death rates for EU13 countries compared with that of EU15 countries, is highly unfavorable. According to the latest available data, the average relative risk of premature death caused by CVDs is 2.86 in the EU13 countries, and it is particularly high in Bulgaria (4.86), Latvia (4.69), Lithuania (4.29), Romania (3.53), and Hungary (3.35) [(World Health Organization (WHO) HFA, 2016)]. Although these figures clearly indicate that the effectiveness of preventive interventions against CVDs is not sufficient in these countries, studies to identify potential targets for prevention measures are missing.

It is generally accepted that in addition to lifestyle-modifying interventions, considerable benefit can be derived from preventive medication. Since hypertension is one of the most robust predictors of CVD risk (Rapsomaniki et al., 2014) antihypertensive treatment for blood pressure reduction is particularly important. Recently it was clearly showed by Vrijens et al. in a paper based on the outcomes of discussions by a European group of experts on the current situation of medication adherence in hypertension that “achieving satisfactory adherence may have far greater impact than any other maneuver to improve antihypertensive treatments, and healthcare systems must evolve to meet this challenge” (Vrijens et al., 2017).

The prevalence of hypertension in different societal groups has been extensively studied (Li et al., 2017), but the relationship between SES and hypertension has been reported with conflicting results. Based on the findings of a systematic search performed in PubMed, ProQuest and Cochrane databases for observational studies on hypertension prevalence and SES, a meta-analysis was carried out. The authors reported an overall increased risk of hypertension among the lowest SES for income, occupation and especially for education (Leng et al., 2015).

The results from studies on the relationship between SES and antihypertensive medication can be interpreted with severe restrictions. The systematic review and meta-analysis on SES and non-adherence to antihypertensive drugs showed (Alsabbagh et al., 2014) that among the 32 studies, only seven examined more than one component while none performed a multidimensional assessment. The pooled adjusted risk estimate for nonadherence according to SES (high vs. low) was 0.89 (0.87–0.92 95% confidence; *P* < 0.001). However, the authors concluded that the individual studies involved in the meta-analysis have not found a strong association between low SES and nonadherence to antihypertensive medications. The authors note that important limitations in the assessment of SES can be identified in virtually all of the studies and emphasize that “future studies are required to ascertain whether a stronger association is observed when SES is determined by comprehensive measures.”

The aim of our present study was to provide data on cardiovascular disease mortality and morbidity as well as types of and adherence (primary non-compliance) to antihypertensive medication in association with the socioeconomic characteristics of various population groups in Hungary. SES was characterized by a multidimensional deprivation index as a composite indicator, which included seven key factors that affect socioeconomic status. Using a conceptually new approach, the relationship between prescription and redemption rates was used to define the contribution of patient and/or physician and/or health system factors to the inefficiency of antihypertensive utilization, if one exists, on CVD prevention. Because physicians in general practice are the key persons that initiate, coordinate, and provide long-term follow-up for CVD prevention (Mauskop and Borden, 2011), our study was performed as a cross-sectional analysis, utilizing data on antihypertensive prescription and redemption rates from all general practices in Hungary.

## Materials and methods

This study focused on the comparative analysis of data regarding prescriptions by general practitioners and redeemed prescriptions for antihypertensive drugs in Hungary during 2012, the last year for which all data that are necessary for a district level analysis were available in the validated databases. The associations of deprivation with the morbidity and mortality caused by diseases of the circulatory system (ICD-10: I00-I99), ischaemic heart diseases (ICD-10: I20-I25), cerebrovascular diseases (ICD-10: I60-I69), and particularly hypertensive diseases (ICD-10: I10-I15) as well as the associations between deprivation and antihypertensive drug utilization (prescription and redemption) were assessed. The relationship between the level of deprivation and the prescribing pattern of antihypertensive drugs was also examined.

### Data

For the year 2012, mortality data were derived from the Hungarian Central Statistical Office, while population data were obtained from the Central Office for Administrative and Electronic Public Services. Both mortality and population data for the districts were stratified by 5-year age bands and sex. Prevalence data were extracted from the database of the National Data Collection Programme (report on the activities of general practitioners and general pediatricians) of the Hungarian Central Statistical Office for the years 2011–2013 by 10-year age bands. In our study, data for the 35–64 age group were used.

The number of prescriptions of antihypertensive drugs and the number of redeemed antihypertensive prescriptions were attained for each primary health care practice for the entire year of 2012 from the National Health Insurance Fund Administration of Hungary. According to Hungarian regulations, general practitioners can prescribe only one type of medicine as a 1-month dose of one prescription for people who are taking long-term medications. Data on the prescribed and redeemed antihypertensive drugs were grouped by commonly used classes, such as diuretics, beta blocking agents, calcium channel blockers, angiotensin converting enzyme (ACE) inhibitors, angiotensin receptor blockers (ARBs) and ACE inhibitors as well as ARBs combined with diuretics (Gu et al., 2012).

### Deprivation index (DI) calculation

A deprivation index (DI) was used to provide information about socioeconomic deprivation compared with the national average for 2011. Socioeconomic indicators for the DI at the municipality level were chosen from available data stored at the Regional Informational System of the Ministry of Local Government and Regional Development. The data were originally obtained from Hungarian Central Statistical Office (Census, 2011) and Hungarian Tax and Financial Control Administration (2011).

The method for calculating DI values and their usefulness in identifying SES-related inequalities in CVD mortality were described previously (Juhász et al., 2010), and it was successfully used in previous studies designed to characterize the association between deprivation and mortality amenable to healthcare (Nagy et al., 2012), between deprivation and premature mortality due to alcoholic liver disease (Nagy et al., 2014) and between deprivation and statin utilization in Hungary (Boruzs et al., 2016). Briefly, the DI is based on seven municipality-level elementary socioeconomic indicators, including income, level of education, rate of unemployment, rate of one-parent families, rate of large families, density of housing and car ownership. The variables were transformed using natural log transformation and standardization (*z*-scores). The municipality-specific index is a weighted sum of the *z*-scores, with higher values representing greater deprivation. The weight of each variable was determined based on the standardized scoring coefficients using a principal component analysis. The areas with positive index values are municipalities with a lower socioeconomic status compared with the national average, and the converse was shown in districts with negative index values.

### Study at the district level

Administratively, Hungary is divided into 19 counties and the capital, Budapest; thus, it has 20 European regions at the third level of the Nomenclature of Territorial Units for Statistics. The counties are further subdivided into 198 districts, which constitute the Local Administrative Units 1 that were formerly known as the Nomenclature of Territorial Units for Statistics level 4 of Hungary (European Commission, 2007).

In Hungary, the number of GP practices operating in 1 781 municipalities was 6,658 in 2012. The size of the practices, such as the number of clients served, varied widely (800–3,000 persons/practice), and the average size was 1,488 persons/practice. Generally, more family practices operate in higher populated municipalities, whereas one family practitioner serves more than one municipality in less populated areas. In addition, there are primary healthcare practitioners with obligations to provide in-area care and those without such obligations. Considering the fact that the free choice of family physicians is a norm in Hungary, that the detailed population data for practices as well as the antihypertensive utilization data by age groups are not available due to personal privacy, and that the DI is not available at the practice level to reduce the risk of misclassification, we aggregated all data for the district level. The deprivation for each district was calculated using the population-weighted average of DI. To define the frequency of prescription and that of redemption, the denominator was the size of the >30-year-old population adjusted by the rate of the >60-year-old population of the district. All districts included in the analysis were classified into 3 groups or tertiles, ranging from the least deprived (tertile I) to the most deprived (tertile III), with each containing a third of the districts analyzed. Grouping the districts into three tertiles could guarantee the robustness of our results even in the case when the sample size is relatively low (number of death caused by hypertensive diseases).

Using the “disease mapping” option within the Rapid Inquiry Facility (RIF) (Beale et al., 2010), spatial patterns of cardiovascular mortality (ICD-10: I00-I99) for the 30–64 age group in 2012 were investigated and visualized at the district level. Hierarchical Bayesian-smoothed indirectly standardized mortality ratios (relative risks) were calculated using Besag, York and Mollie's model with the INLA method (Besag et al., 1991; Rue et al., 2009) with the expected cases based on the age-specific death rates for the Hungarian population. The maps for each health outcome show the relative risks and exceedance posterior probabilities (Richardson et al., 2004).

The relative frequency of prescription for the selected drugs was also mapped for the >30-year-old population using RIF. The associations among premature mortalities, prevalence of diseases and the frequency of antihypertensive prescriptions by agents as well as redeemed antihypertensive prescriptions by agents were defined using tertiles of DI as a district-based categorical covariate and the “risk analysis” capabilities of the RIF. Chi-square tests for homogeneity and linear trend were also performed to test the global associations of DI with morbidity, mortality and antihypertensive utilization.

The ratios between the number of redeemed prescriptions and that of the prescriptions for antihypertensive drugs were used to characterize the level of primary non-compliance. The results were analyzed by types of drugs and deprivation tertiles.

## Results

### The spatial distribution of premature mortality and the prevalence of selected cardiovascular diseases as well as their association with deprivation

**Figure 1:**
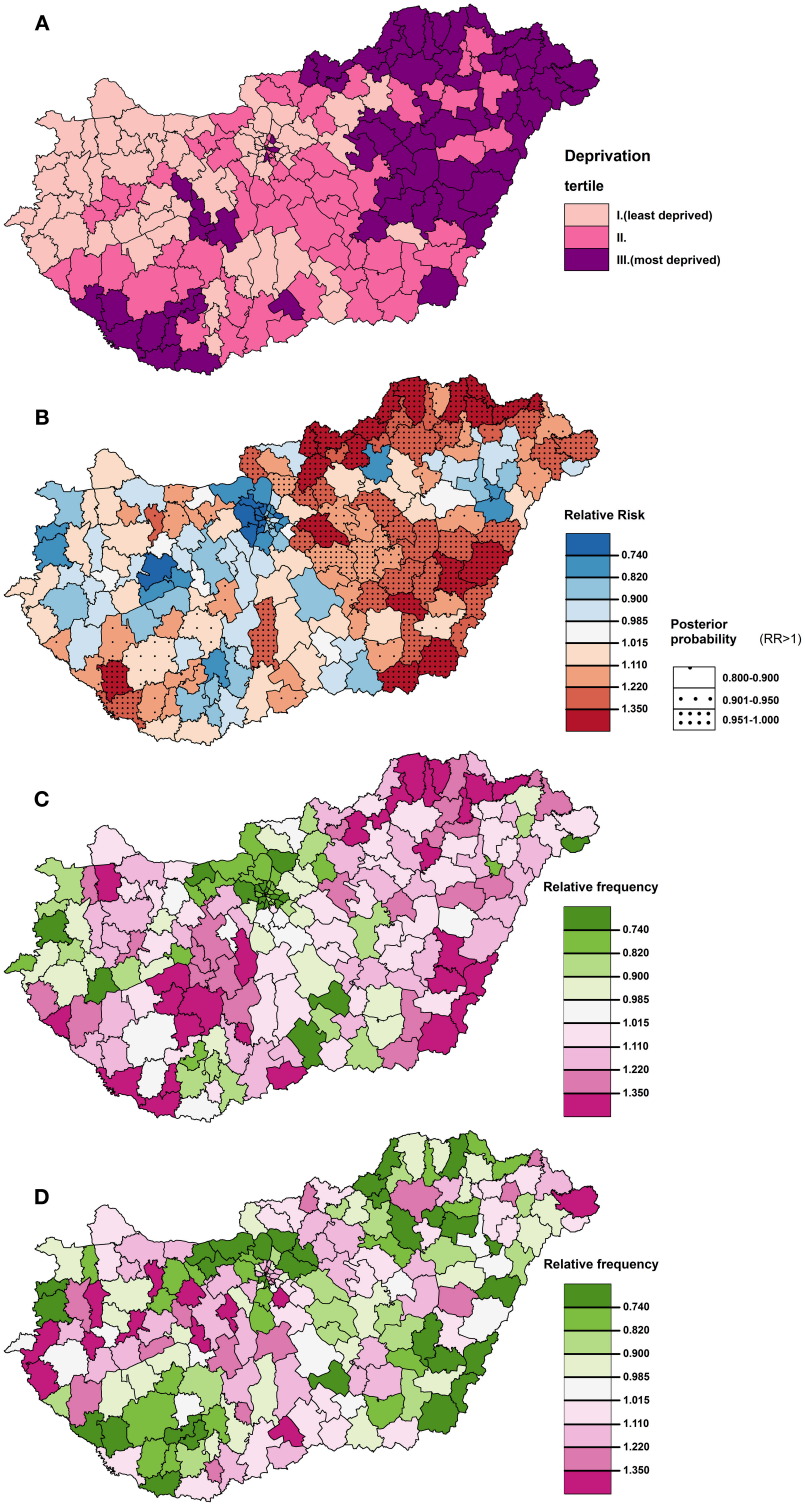
The spatial distribution of deprivation **(A)** and premature mortality due to diseases of the circulatory system in the 30–64 age group **(B)**, and that of relative frequencies of prescription for ACE inhibitors **(C)** and ARBs **(D)** in the >30 age group at district level in 2012 in Hungary.

Deprivation index values defined by districts varied widely from −3.76 to +5.83, which indicates a high level of socioeconomic inequalities in the country. The tertiles based on the DI values were defined as ranges of −3.76 ≤ DI ≤ −0.6 with an average of −1.32 (tertile I); −0.6 < DI ≤ 0.58 with an average of −0.04 (tertile II); and 0.58 < DI ≤ 5.83 with an average of 1.59. The distribution of the DI values shows that the least-favored districts were found in the northeastern and southwestern part of Hungary in 2011. The least deprived districts of the country were localized at the northwestern region of Hungary, in the capital city of Budapest and its neighboring areas (Figure 1A). The spatial distribution of premature mortality due to diseases of the circulatory system in Hungary was characterized by significant inequalities, the areas of highest SMRs were found in the eastern part of the country, and along the north-eastern border of Hungary (Figure 1B).

**Figure 2:**
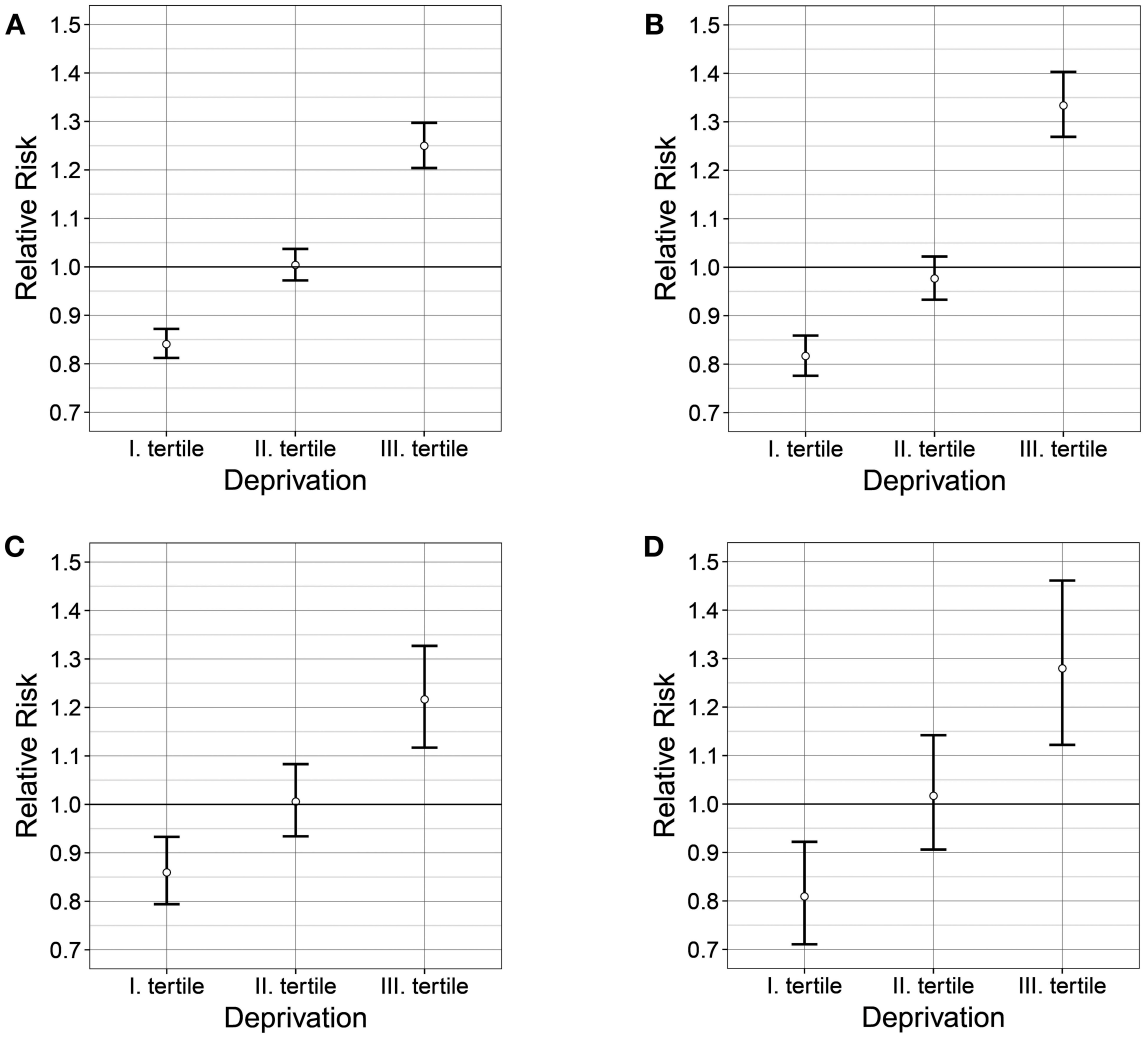
Relationship between the deprivation and the relative risk of premature mortalities caused by diseases of the circulatory system **(A)** chronic ischaemic heart diseases **(B)**, cerebrovascular diseases **(C)**, and hypertensive diseases **(D)** in 2012 in Hungary.

The resultant pattern of excess risks caused by the examined causes of premature mortality showed a correlation with the spatial pattern of deprivation. The results of the risk analysis showed a significant association between the risk of premature cardiovascular mortality and deprivation (χ^2^ homogeneity = 229.21, *P* = 0, χ^2^ linearity = 226.91, *P* = 0); between the risk of cerebrovascular premature mortality and deprivation (χ^2^ homogeneity = 33.38, *P* = 0, χ^2^ linearity = 33.12, *P* = 0); between the risk of chronic ischaemic heart diseases and deprivation (χ^2^ homogeneity = 191.1 *P* = 0, χ^2^ linearity = 182.58, *P* = 0); and between the risk of hypertensive diseases and deprivation (χ^2^ homogeneity = 23.8 *P* = 0, χ^2^ linearity = 23.7, *P* = 0). Premature mortality in the areas of the highest deprivation tertile exceeded the national average mortality by 25% for mortality due to diseases of the circulatory system, by 22% for mortality due to cerebrovascular disease, and by 28%—with a wider confidence interval - for mortality due to hypertensive diseases (Table 1, Figure 2).

**Table 1:** Relative risks of premature mortality due to diseases of the circulatory system, chronic ischaemic heart diseases, cerebrovascular diseases and hypertensive diseases for 30–64 age group at the district level in Hungary in 2012 by DI tertiles.

**DI tertiles**	**Mortality due to diseases of the circulatory system (ICD-10.: I00-I99)**	**Mortality due to chronic ischaemic heart diseases (ICD-10.: I20-I25)**	**Mortality due to cerebrovascular diseases (ICD-10.: I60-I69)**	**Mortality due to hypertensive diseases (ICD-10.: I10-I15)**
	**Relative risk (95% CI)**	**Relative risk (95% CI)**	**Relative risk (95% CI)**	**Relative risk (95% CI)**
I. (least deprived)	0.841 [0.812–0.872]	0.817 [0.776–0.859]	0.860 [0.794–0.933]	0.809 [0.711–0.922]
II.	1.004 [0.972–1.037]	0.977 [0.933–1.022]	1.006 [0.934–1.083]	1.017 [0.906–1.142]
III. (most deprived)	1.250 [1.204–1.297]	1.334 [1.269–1.403]	1.217 [1.117–1.327]	1.280 [1.122–1.461]

**Figure 3:**
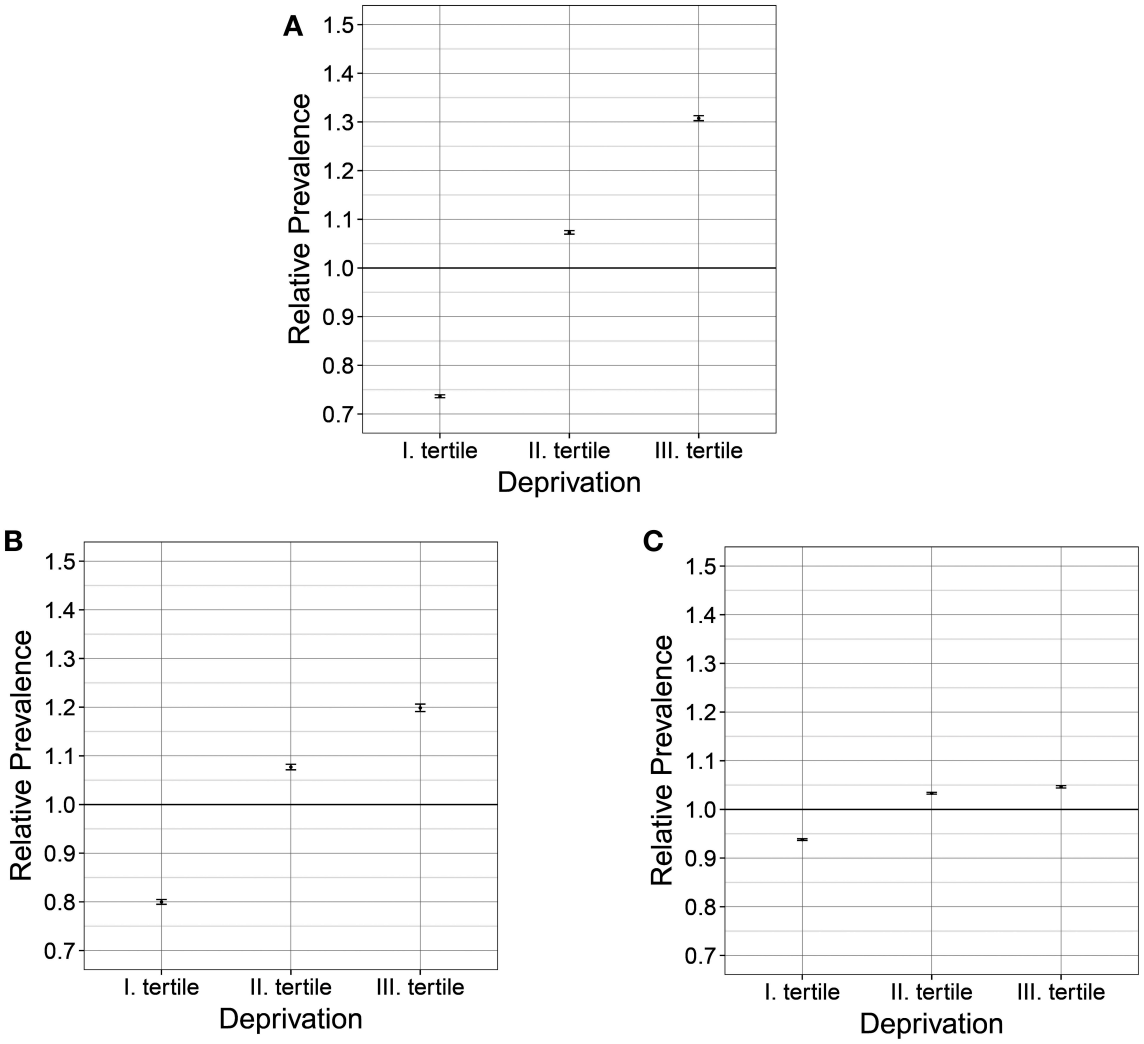
Relationship between the deprivation and the relative prevalence of chronic ischaemic heart diseases **(A)**, cerebrovascular diseases **(B)** and hypertensive diseases **(C)** in 2011–2013 in Hungary.

Regarding the relative prevalence values, the results of the risk analysis also showed a significant association between the relative prevalence of chronic ischaemic heart diseases and deprivation (χ^2^ homogeneity = 41872.22, *P* = 0, χ^2^ linearity = 41072.6, *P* = 0); between the relative prevalence of cerebrovascular diseases and deprivation (χ^2^ homogeneity = 8823.8, *P* = 0, χ^2^ linearity = 8241.2, *P* = 0); and between the relative prevalence of hypertensive diseases and deprivation (χ^2^ homogeneity = 7085.9 *P* = 0, χ^2^ linearity = 5755.9, *P* = 0) (Figures 3A–C). For hypertensive diseases, fewer differences were found in morbidity associated with deprivation. In the areas of the highest deprivation tertile, morbidity exceeded the national average by only 4% (Table 2, Figure 3).

**Table 2:** Relative prevalence of chronic ischaemic heart diseases, cerebrovascular diseases and hypertensive disease for 35–64 age group at the district level in Hungary in 2012 by DI tertiles.

**DI tertiles**	**Prevalence of chronic ischaemic heart diseases (ICD-10.: I20-I25)**	**Prevalence of cerebrovascular diseases (ICD-10.: I60-I69)**	**Prevalence of hypertensive diseases (ICD-10.: I10-I15)**
	**Relative prevalence (95% CI)**	**Relative prevalence (95% CI)**	**Relative prevalence (95% CI)**
I. (least deprived)	0.736 [0.733–0.739]	0.800 [0.795–0.805]	0.938 [0.936–0.940]
II.	1.073 [1.070–1.077]	1.077 [1.071–1.083]	1.033 [1.031–1.035]
III. (most deprived)	1.308 [1.303–1.313]	1.199 [1.191–1.206]	1.046 [1.044–1.049]

### Antihypertensive drug utilization (prescription and redemption rates)

In Hungary, altogether, 41,221,460 antihypertensive regimens were prescribed in 2012, and only 64.85% of those (26,732,297) were redeemed. The frequency of prescription was 6.111 [6.108–6.112] and the frequency of redemption was 3.963 [3.961–3.964] per person aged >30- year-old (Table 3).

**Table 3:** Antihypertensive utilization (prescription, redemption and redemption rate) by drug types in Hungary in 2012.

**Drug types**	**Total number of prescription**	**Total number of redemption**	**Frequency of prescription**	**Frequency of redemption**	**Redemption rate (%)**
			**(per person aged 30**+ **years)**		
Antihypertensives (all)	41,221,460	26,732,297	6.111	3.963	64.85
Beta blocking agents	10,136,446	7,260,132	1.503	1.076	71.62
ACE inhibitors	7,306,211	5,169,763	1.083	0.766	70.76
ACE inhibitors in combination with diuretics	6,284,235	2,919,612	0.932	0.433	46.46
Calcium channel blockers	6,228,901	4,432,300	0.923	0.657	71.16
Diuretics	5,857,905	4,073,469	0.868	0.604	69.54
ARBs	2,750,115	1,908,731	0.408	0.283	69.41
ARBs in combination with diuretics	2,657,647	968,290	0.394	0.144	36.43

*ACE, Angiotensin converting enzyme; ARB, Angiotensin receptor blocker*.

The redemption rate of different types of antihypertensive drugs differed significantly. The redemption rates of beta blocking agents, ACE inhibitors, calcium channel blockers, diuretics, and ARBs were approximately 70%, while the redemption rates of ACE inhibitors and ARBs combined with diuretics were significantly lower (46.46 and 36.43%, respectively). Furthermore, the lowest frequencies of prescription and redemption per person aged >30-year-old were found for ARBs combined with diuretics, and the highest frequencies were shown for beta blocking agents (Table 3).

**Figure 4:**
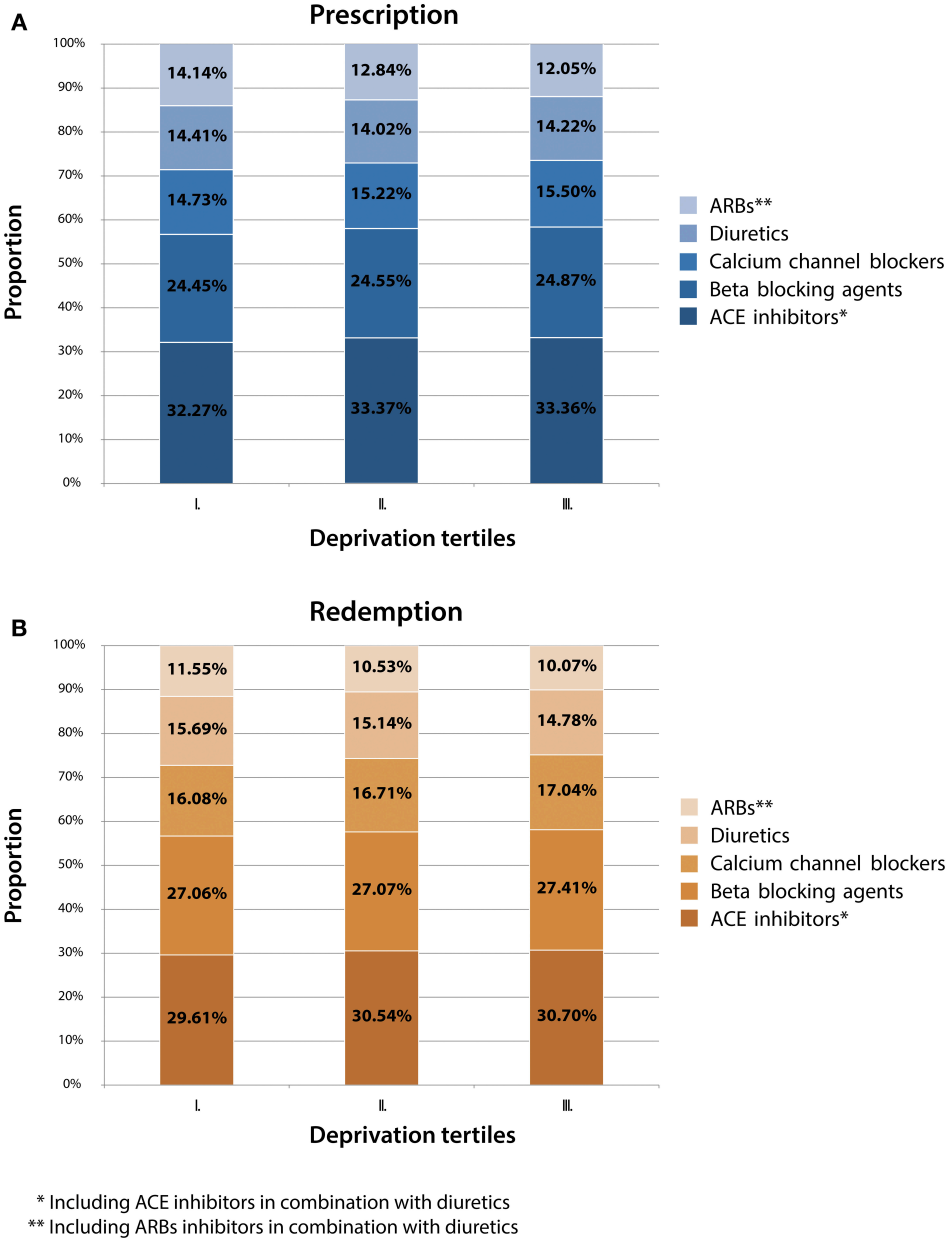
Antihypertensive drug prescription **(A)** and redemption **(B)** by Deprivation Index tertiles and drug types in Hungary in 2012.

Proportion of ACE inhibitor (with and without diuretics) prescription and redemption was the highest (about one third of the total) in each deprivation group (tertiles I-III.), while the prescription and redemption of beta blocking agents was the second highest, and the prescription and redemption of calcium channel blockers was the third highest (Figures 4A,B, Table 4). Representation of these drugs' prescription and redemption increased by deprivation level; proportions of ARB prescription in the most deprived tertile were lower (by approximately 2%) than that in the least deprived tertile (12.05 vs. 14.14%). The same trend was observed in case of ARB redemption (its representation in the most deprived tertile: 10.07%; and in the least deprived tertile: 11.55%) (Figure 3, Table 4).

**Table 4:** Antihypertensive drug utilization by types of drugs and DI tertiles in Hungary in 2012.

	**DI tertiles**		
	**I. (Least deprived)**	**II**.	**III. (Most deprived)**
**Drug types**	**Prescription**		
	**Totals (number) and proportions in percentage (%) [95% CI]**		
ACE inhibitors^*^	4,818,107	5,388,432	3,383,907
	32.27 [32.24–32.29]	33.37 [33.35–33.4]	33.36 [33.33–33.39]
Beta blocking agents	3,650,127	3,963,420	2,522,899
	24.45 [24.42–24.47]	24.55 [24.53–24.57]	24.87 [24.85–24.9]
Calcium channel blockers	2,199,870	2,457,276	1,571,755
	14.73 [14.71–14.75]	15.22 [15.20–15.24]	15.5 [15.47–15.52]
Diuretics	2,152,211	2,263,549	1,442,145
	14.41 [14.40–14.43]	14.02 [14.00–14.04]	14.22 [14.20–14.24]
ARBs^**^	2,111,313	2,073,681	1,222,768
	14.14 [14.12–14.16]	12.84 [12.83–12.86]	12.05 [12.03–12.07]
	**Redemption**		
	**Totals (number) and proportions in percentage [95% CI]**		
ACE inhibitors^*^	2,757,054	3,163,058	2,169,263
	29.61 [29.58–29.64]	30.54 [30.51–30.57]	30.70 [30.67–30.73]
Beta blocking agents	2,519,785	2,803,597	1,936,750
	27.06 [27.04–27.09]	27.07 [27.04–27.10]	27.41 [27.38–27.44]
Calcium channel blockers	1,497,488	1,730,739	1,204,073
	16.08 [16.06–16.11]	16.71 [16.69–16.73]	17.04 [17.01–17.07]
Diuretics	1,460,810	1,568,174	1,044,485
	15.69 [15.67–15.71]	15.14 [15.12–15.16]	14.78 [14.76–14.81]
ARBs^**^	1,075,182	1,090,599	711,240
	11.55 [11.53–11.57]	10.53 [10.51–10.55]	10.07 [10.04–10.09]

* *, including ACE inhibitors in combination with diuretics*;

** *, including ARBs in combination with diuretics; ACE, Angiotensin converting enzyme; ARB, Angiotensin receptor blocker*.

The spatial distribution of deprivation and of the relative frequencies of prescription of the selected antihypertensive drug prescriptions (ACE inhibitors with and without diuretics, calcium channel blockers and ARBs with and without diuretics) are shown at the district level in Hungary in Figure 4. Areas of the highest deprivation showed high frequencies of ACE inhibitor (Figures 1A,C) in midwestern, northeastern, eastern and southwestern parts of Hungary. However, in those parts of the country, low relative frequencies of prescriptions for ARBs were found (Figures 1A,D), while in midswestern and northwestern part of Hungary was associated with high relative frequency values.

The results of the risk analysis showed a linear association of the relative frequency of prescriptions for almost every drug with its redemption and deprivation. However, varying associations were found regarding ARBs and ARBs in combination with diuretics (Table 5).

**Table 5:** Relative frequency of the prescription and redemption of antihypertensive drugs at the district level in Hungary in 2012 by DI tertiles.

	**DI tertiles**		
	**I. (Least deprived)**	**II**.	**III. (Most deprived)**
	**Prescription**		
**Drug types**	**Relative frequency [95% CI]**		
Antihypertensives (all)	0.9377 [0.9372–0.9382]	1.0201 [1.0196–1.0206]	1.0711 [1.0704–1.0717]
Beta blocking agents	0.9338 [0.9329–0.9348]	1.0166 [1.0156–1.0176]	1.0833 [1.0819–1.0846]
ACE inhibitors	0.9253 [0.9240–0.9260]	1.0386 [1.0370–1.0400]	1.0610 [1.0590–1.0630]
ACE inhibitors in combination with diuretics	0.9139 [0.9127–0.9151]	1.0222 [1.0210–1.0235]	1.1064 [1.1047–1.1081]
Calcium channel blockers	0.9168 [0.9155–0.9180]	1.0257 [1.0245–1.0270]	1.0963 [1.0946–1.0980]
Diuretics	0.9481 [0.9469–0.9494]	1.0088 [1.0075–1.0102]	1.0728 [1.0711–1.0746]
ARBs	0.9988 [0.9969–1.0007]	1.0205 [1.0186–1.0224]	0.9677 [0.9653–0.9702]
ARBs in combination with diuretics	1.0040 [1.0021–1.0059]	0.9889 [0.9870–0.9909]	1.0116 [1.0091–1.0142]
	**Redemption**		
	**Relative frequency [95% CI]**		
Antihypertensives (all)	0.9043 [0.9037–0.9049]	1.0073 [1.0066–1.0079]	1.1480 [1.1471–1.1488]
Beta blocking agents	0.9029 [0.9018–0.9040]	1.0020 [1.0009–1.0032]	1.1588 [1.1572–1.1604]
ACE inhibitors	0.8885 [0.8872–0.8898]	1.0259 [1.0245–1.0273]	1.1429 [1.1410–1.1448]
ACE inhibitors in combination with diuretics	0.8810 [0.8793–0.8828]	0.9991 [0.9973–1.0009]	1.2000 [1.1974–1.2027]
Calcium channel blockers	0.8799 [0.8785–0.8813]	1.0133 [1.0118–1.0148]	1.1776 [1.1755–1.1797]
Diuretics	0.9263 [0.9248–0.9278]	1.0047 [1.0031–1.0063]	1.1165 [1.1143–1.1186]
ARBs	0.9656 [0.9633–0.9678]	1.0089 [1.0066–1.0112]	1.0430 [1.0400–1.0460]
ARBs in combination with diuretics	0.9656 [0.9625–0.9687]	0.9508 [0.9476–0.9539]	1.1399 [1.1355–1.1443]

*ACE, Angiotensin converting enzyme; ARB, Angiotensin receptor blocker*.

Reverse J-shaped association was detected between the relative frequency of ARB prescriptions and deprivation. In the highest deprivation tertile, it was lower than the national average by 3% (relative frequency: 96.77% [96.53–97.02]) (Table 5). The relative frequency of the prescriptions and redemptions for ARBs in combination with diuretics showed a J-shaped association with deprivation.

The frequency of prescription for all antihypertensive drugs in the lowest tertile (i.e., in the least deprived tertile) was 5.7273 [5.7244–5.7302], which was 7% (relative frequency: 93% [93.72–93.82]) lower than the Hungarian average, whilst the frequency in tertile III (i.e., in the most deprived tertile) was 6.5809 [6.5769–6.5850], which exceeded the national average by 7% (relative frequency: 107% [107.04–107.17]) (Table 6). Furthermore, the frequency of redemption for all antihypertensive drugs in the lowest tertile was 3.6038 [3.6015–3.6061], which was 10% lower than the national average (relative frequency: 90% [90.37–90.49]), and in the highest tertile, the frequency was 4.6047 [4.6013–4.6081], which was 15% higher (relative frequency: 15% [114.71–114.88]) than the national average (Table 6). Similar linear associations were found in the relative frequency for almost every drug, except the ARBs in combination with diuretics prescription.

**Table 6:** Frequency of the prescription and redemption of antihypertensive drugs per person aged 30+ years, at the district level in Hungary in 2012 by DI tertile.

	**DI tertiles**		
	**I. (least deprived)**	**II**.	**III. (most deprived)**
	**Prescription**		
**Drug types**	**Frequency per person aged 30**+ **years [95% CI]**		
Antihypertensives (all)	5.7273 [5.7244–5.7302]	6.2766 [6.2735–6.2796]	6.5809 [6.5769–6.5850]
Beta blocking agents	1.4062 [1.4048–1.4077]	1.5408 [1.5392–1.5423]	1.6320 [1.6300–1.6340]
ACE inhibitors	1.0050 [1.0037–1.0062]	1.1341 [1.1328–1.1354]	1.1673 [1.1656–1.1691]
ACE inhibitors in combination with diuretics	0.8535 [0.8531–0.8539]	0.9606 [0.9604–0.9609]	1.0417 [1.0401–1.0433]
Calcium channel blockers	0.8428 [0.8423–0.8432]	0.9529 [0.9527–0.9532]	1.0161 [1.0146–1.0177]
Diuretics	0.8215 [0.8210–0.8220]	0.8768 [0.8764–0.8772]	0.9305 [0.9301–0.9309]
ARBs	0.4067 [0.4061–0.4073]	0.4163 [0.4157–0.4169]	0.3951 [0.3943–0.3959]
ARBs in combination with diuretics	0.3992 [0.3986–0.3998]	0.3869 [0.3863–0.3875]	0.3980 [0.3972–0.3988]
	**Redemption**		
	**Frequency per person aged 30**+ **years [95% CI]**		
Antihypertensives (all)	3.6038 [3.6015–3.6061]	4.0398 [4.0373–4.0422]	4.6047 [4.6013–4.6081]
Beta blocking agents	0.9802 [0.9790–0.9814]	1.0926 [1.0913–1.0938]	1.2585 [1.2568–1.2603]
ACE inhibitors	0.6869 [0.6864–0.6875]	0.7972 [0.7967–0.7977]	0.8949 [0.8945–0.8954]
ACE inhibitors in combination with diuretics	0.3776 [0.3771–0.3782]	0.4348 [0.4342–0.4354]	0.5291 [0.5283–0.5299]
Calcium channel blockers	0.5716 [0.5710–0.5722]	0.6704 [0.6698–0.6710]	0.7817 [0.7810–0.7823]
Diuretics	0.5576 [0.5570–0.5582]	0.6074 [0.6068–0.6080]	0.6764 [0.6757–0.6772]
ARBs	0.2719 [0.2713–0.2724]	0.2856 [0.2851–0.2862]	0.2982 [0.2974–0.2989]
ARBs in combination with diuretics	0.1390 [0.1386–0.1394]	0.1373 [0.1369–0.1377]	0.1656 [0.1651–0.1663]

*ACE, Angiotensin converting enzyme; ARB, Angiotensin receptor blocker*.

When the number of redeemed prescriptions and that of the prescriptions for antihypertensive drugs were compared, the redemption rate was found to be higher as the deprivation became more pronounced, i.e., better compliance was observed in tertile II than in tertile I, and the highest rates (independent from the types of drugs) were always associated with the districts with the highest deprivation (Table 7).

**Table 7:** Redemption rate of antihypertensive drugs by DI tertiles at the district level in Hungary in 2012.

	**DI tertiles**		
	**I. (Least deprived)**	**II**.	**III. (Most deprived)**
**Drug types**	**Redemption rate (%) [95% CI]**		
Antihypertensives (all)	62.35 [62.33–62.38]	64.14 [64.12–64.16]	69.66 [69.63–69.69]
Beta blocking agents	69.39 [69.33–69.44]	70.84 [70.79–70.88]	76.27 [76.21–76.33]
ACE inhibitors	68.11 [68.05–68.18]	70.10 [70.04–70.15]	75.77 [75.70–75.84]
ACE inhibitors in combination with diuretics	44.84 [44.77–44.90]	45.34 [45.28–45.40]	50.42 [50.34–50.49]
Calcium channel blockers	68.07 [68.01–68.13]	70.43 [70.38–70.49]	76.61 [76.54–76.67]
Diuretics	67.87 [67.81–67.94]	69.28 [69.22–69.34]	72.43 [72.35–72.50]
ARBs	67.17 [67.07–67.27]	68.97 [68.88–69.06]	74.71 [74.58–74.83]
ARBs in combination with diuretics	35.04 [34.94–35.14]	35.29 [35.20–35.39]	41.20 [41.06–41.34]

*ACE, Angiotensin converting enzyme; ARB, Angiotensin receptor blocker*.

## Discussion

Inequalities in mortality and morbidity between populations with different (lower and higher) socioeconomic positions are widely described in the literature with a special focus on different regions of Europe (see reviewed in Mackenbach et al., 2017). de Gelder et al. have analyzed trends in socioeconomic inequalities in mortality over four decades in six European countries among them Hungary and provided a first analysis of long-term trends in inequalities in mortality in Central/Eastern Europe (de Gelder et al., 2017) where history had a profound impact on mortality and life expectancy (Mackenbach, 2013). They showed that relative inequalities in all-cause mortality generally increased, but more so in Hungary and Norway than elsewhere and absolute inequalities often narrowed but went up in these two countries. In general, Central and Eastern European countries are in which inequalities in mortality have “disastrously exploded” since the early 1990s (Mackenbach, 2017).

The East-West life expectancy gap in Europe has been a well-known epidemiological phenomenon for a long time (Jakovljevic et al., 2016a). It was clearly demonstrated that the difference of extraordinary magnitude is most strongly related to the high rate of premature mortality from cardiovascular causes (Hertzman and Bobak, 1996). The question whether premature mortality is the current Iron Curtain in Europe (Zatonski et al., 2016) seems to be reasonable. The inequalities in CVD mortality and that in preventive medication targeting CVD risk factors are very intensely studied in the populations of the high-income EU15 countries (Alsabbagh et al., 2014; Leng et al., 2015), but reports for the EU13 countries, especially for the most affected Central, Eastern and Southeastern European (CESEE) EU member states, are extremely scarce (Jakovljevic et al., 2015), and most of them are methodologically inappropriate.

Regarding the relationship between SES and CVDs from CESEE countries, only our group published a methodological report on the development of a deprivation index (DI) to characterize SES with a multidimensional composite indicator, in which not only information about socioeconomic deprivation in Hungary was provided but also a statistically significant association between SES characterized by DI and the spatial distribution of premature mortality due to diseases of the circulatory system for the years 1998–2004 was also shown (Juhász et al., 2010). The analysis was carried out to demonstrate using the example of cardiovascular diseases that inequalities in socioeconomic status may reflect the spatial distribution of health status in a population. Our findings on the distribution of deprivation and CVD mortality and morbidity corroborate this hypothesis.

Concerning the relationship between SES and preventive medication to reduce CVD risk, our recent publication on the association between statin utilization and socioeconomic deprivation in Hungary (Boruzs et al., 2016) is the only publication from CESEE countries. In this country-wide analysis, the standardized premature CVD mortality rates, frequency of statin prescriptions, redeemed statin prescriptions, and ratios for compliance compared to the national average were mapped, and their associations with deprivation (tertile of deprivation index as a district-based categorical covariate) were defined. The risk analysis showed a significant positive association between deprivation and the relative risk of premature cardiovascular mortality and a reverse J-shaped association between the relative frequency of statin prescriptions and deprivation. Districts with the highest deprivation showed a low relative frequency of statin prescriptions; however, significantly higher primary compliance (redemption) was observed in these districts. Our data clearly suggest that insufficient statin utilization may represent a significant barrier to reducing CVD mortality, particularly among people living in highly deprived areas of the country. Nevertheless, it is worth mentioning that in Hungary the high prevalence of health behavior risk factors (as smoking, uncontrolled alcohol consumption, physical inactivity, unhealthy nutrition, etc.) is a severe problem regarding the prevention of chronic diseases (Sándor et al., 2017). Among Roma heavy smoking and unhealthy diet were 1.5–3 times more prevalent than in the general Hungarian population (Kósa et al., 2007; Sándor et al., 2017). The lower prevalence of healthy lifestyle activities among lower educated, lower income and elder people living in highly deprived small settlements was observed (Paulik et al., 2010, 2011). Several policies (anti-smoking legislation, new taxes on “unhealthy” food as sweetened, salty and fatty products) were introduced by the Hungarian government to facilitate favorable changes in health behaviors, but in all probability quadrupling of public works expenditure in the period between 2010 and 2015 had the most profound effect (Sándor et al., 2017). In the period between 2009 and 2013 the unemployment rate in Hungary was above 10%, which—by introducing the public work program—fell to 6.6% in 2015 (World Health Organization. European health information gateway, 2017).

Our present study was designed to investigate the field of antihypertensive utilization and its association with the socioeconomic characteristics of various population groups in Hungary by focusing on the comparative analysis of data for prescriptions by general practitioners and the redeemed prescriptions for antihypertensive drugs at the district level during 2012. In addition, investigations on the relationship between deprivation and premature mortality caused by different CVDs (among them hypertension) as well as between deprivation and their prevalence were also carried out.

To the best of our knowledge, this is the first comprehensive study that analyzed the relationship of SES with CVD mortality, morbidity, and the utilization of antihypertensive drugs in a CESEE country characterized by an extremely high relative risk of premature CVD mortality using a multidimensional composite indicator to properly characterize the SES and using risk analysis capabilities to estimate the relationships between deprivation and CVD burden as well as antihypertensive medication. In complexity, this study is unique among the previously published studies in this field from different countries around the world.

Risk analysis showed a significant association of the risk of premature cardiovascular and cerebrovascular mortality with deprivation. The spatial pattern of prevalence values for different diseases was similar to the distribution of their relative risk of mortality; however, inequality was less dramatic for hypertensive diseases. Based on the fact that the prevalence of hypertension exceeded the national average by only 4% in the areas of the highest deprivation tertile, while the prevalence of chronic ischaemic heart diseases and cerebrovascular diseases was higher than the national average by approximately 30% and 20%, respectively, it is reasonable to suppose that in a significant number of cases, hypertension remains unknown until the manifestation of its complications, i.e., late diagnosis. This assumption is supported by the findings that the frequency of prescriptions for all antihypertensive drugs is 7% higher than the national average, while the frequency of redemption is 15% higher than the national average in the regions with the highest deprivation. The higher frequency of prescriptions may indicate that fewer hypertensive patients can be controlled on monotherapy, i.e., more patients require more than one anti-hypertensive medication for blood pressure control, while the significantly lower level of primary non-compliance may indicate that the patients take medical advice when symptoms of hypertensive crisis appear or in comorbid conditions.

The patterns of antihypertensive drugs prescription and redemption significantly differ by DI tertile; although the differences expressed in a percentage seem to be small, they are statistically significant due to the large number of observations. In areas of the highest deprivation, higher relative frequencies of ACE inhibitor, beta blocker and calcium channel blocker prescriptions and lower relative frequency of ARB prescriptions were found. The representation of ARBs among the antihypertensive medications used was increased with the improvement of socioeconomic status. These findings concur with the observation of a registry-based study on the types of antihypertensive medications used in ambulatory settings in Finland (Härkönen et al., 2015) that high income patients were more likely to use ARBs, while low income patients were more likely to use beta blockers and ACE inhibitors.

Concerning the history of hypertension treatment (Saklayen and Deshpande, 2016) from the discovery of blood pressure lowering effect of thiazides and thiazide-like compounds (Freis et al., 1958) more and more potential pharmacological targets became known and these findings lead to the development and introduction of novel antihypertensive medicines. The milestones on this process are the multicentre randomized placebo-controlled clinical trials—from VA (1967) through TOMHS (1993); ALLHAT (2002) and ASCOT (2005)—among others—to SPRINT (2015) - which were designed not only to clinically test some newly discovered drugs, but also to characterize and compare their effects on CVD morbidity and mortality. The recently completed SPRINT study showed huge reduction in mortality (25%) and CVD events (30%) in the group with systolic blood pressure goal of 120 mmHg (SPRINT Research Group, 2015). Large randomized clinical trials have provided strong evidence that antihypertensive treatment substantially reduces the risk of overall mortality, especially that of cardiovascular diseases as stroke and coronary heart diseases (Chobanian et al., 2003; Turnbull et al., 2008). On the basis of results obtained in meta-analysis of data published on the effect of antihypertensive treatment on secondary prevention of CVD events and all-cause mortality among persons without clinically defined hypertension it could be concluded that even among patients with clinical history of CVD but without hypertension, antihypertensive treatment is associated with decreased risk of stroke, congestive heart failure, composite CVD events, and all-cause mortality (Thompson et al., 2011).

Different types of antihypertensive drugs show different effectiveness for the treatment of hypertension. When perindopril as a new ACE inhibitor drug was introduced among patients with stable coronary heart disease it was showed in the EUROPA study that perindopril treatment can significantly improve outcome, namely it results in a 20% additional risk reduction (Fox and EURopean trial On reduction of cardiac events with Perindopril in stable coronary Artery disease Investigators, 2003). The study group concluded that “treatment with perindopril, on top of other preventive medications, should be considered in all patients with coronary heart disease.” Angiotensin II receptor blockers (ARBs) as “recent advances in risk reduction” were interpreted on the basis of results obtained in the ONTARGET trial (Guthrie, 2009) in which the ACE inhibitor ramipril and the ARB telmisartan were found equally effective in reducing the incidence of cardiovascular death, myocardial infarction, stroke, and hospitalization for heart failure in patients at high risk for cardiovascular disease. In spite of new and new drug discoveries a large scale study designed to compare the effectiveness of thiazide diuretic (TD), ACE inhibitors, ARBs and calcium channel blocker (CCB) monotherapies for the treatment of hypertension on 565,009 patients the authors found that patients who took TDs experienced a lower risk of clinical events compared with patients who took ACE inhibitors, ARBs and CCBs. These results provide a strong rationale for choosing TDs as first-line monotherapy for the control of hypertension (Machado et al., 2017).

In a carefully designed meta-analysis of publications on the relationship between SES and hypertension, Leng et al. could identify only a single publication from the CESEE countries, and even this report lacked considerable merit (Leng et al., 2015). This single study was carried out among central Slovakian adults with a small sample size of 100 probands (50 males and 50 females) in which only 14 participants with low SES were identified; among them, hypertension was found to be less frequent than in the group of participants with high SES (*n* = 15). Concerning the sample size, this finding could not be considered substantial even if the authors themselves did not report, in the very same paper, a conflicting result; namely, when the group was stratified by educational level, the prevalence of hypertension was found to be highest in the least educated group (Hujova and Rostakova, 2013). Studies on Roma, the most disadvantaged ethnic population of the European Union that is accumulating in the CESEE countries, did not reveal significant differences by ethnicity regarding hypertension between Roma and the host populations (Vozarova de Courten et al., 2003; Babinska et al., 2013; Kósa et al., 2015) in Slovakia and Hungary, but the prevalence of hypertension in the Bayash Roma population was significantly lower than what is usually reported for the general population of Croatia (Zeljko et al., 2008). Only a single report mentions a higher prevalence of hypertension among Slovakian Roma than in the general population (Dolinska et al., 2007).

There are some limitations that need to be considered in the interpretation of our findings. In our ecological study the associations detected by the investigation do not prove a causal relationship. The deprivation associated with mortality and preventive medication at the population level may not necessarily be associated with mortality and preventive medication in individuals (Morgenstern, 1995). All of the factors (related to patients, physicians, and health system) that have an effect on antihypertensive utilization cannot be covered in a single study. The effects associated with socioeconomic factors may be mediated by other factors that were not included in our analyses (Radevic et al., 2016). Health system factors were only partially studied, and access to care requires particular attention in future studies (Jakovljevic et al., 2016b). Information about various elements that may influence a patient's likelihood to take antihypertensive medications should also be collected to understand the very low relative redemption rate in case of ARBs and ACE inhibitors when combined with diuretics.

## Ethics statement

Human and animal subjects were not involved in the study, ethical approval was not needed to the study.

## Author contributions

KBo contributed to conception, design, analysis, interpretation the study and drafted manuscript. AJ and CN contributed to design, acquisition, analysis, interpretation and drafted manuscript. ZS, MJ, and KBí contributed to conception, interpretation and critically revised the manuscript. RÁ contributed to conception, design, acquisition, analysis, interpretation and critically revised the manuscript. All authors approved the final manuscript.

### Conflict of interest statement

The authors declare that the research was conducted in the absence of any commercial or financial relationships that could be construed as a potential conflict of interest.

## Abbreviations

ACE: angiotensin converting enzyme
ARB: angiotensin receptor blocker
CESEE: Central, Eastern and Southeastern Europe
CVD: cardiovascular disease
DI: deprivation index
RIF: Rapid Inquiry Facility
SES: socioeconomic status.
